# Diverse architectural properties of Sso10a proteins: Evidence for a role in chromatin compaction and organization

**DOI:** 10.1038/srep29422

**Published:** 2016-07-11

**Authors:** Rosalie P. C. Driessen, Szu-Ning Lin, Willem-Jan Waterreus, Alson L. H. van der Meulen, Ramon A. van der Valk, Niels Laurens, Geri F. Moolenaar, Navraj S. Pannu, Gijs J. L. Wuite, Nora Goosen, Remus T. Dame

**Affiliations:** 1 Leiden Institute of Chemistry, Cell Observatory and Centre for Microbial Cell Biology, Leiden University, Einsteinweg 55, 2333 CC Leiden, The Netherlands; 2 Department of Physics and Astronomy, VU University, Boelelaan 1081, 1081 HV Amsterdam, The Netherlands

## Abstract

Sso10a proteins are small DNA-binding proteins expressed by the crenarchaeal model organism *Sulfolobus solfataricus*. Based on the structure of Sso10a1, which contains a winged helix-turn-helix motif, it is believed that Sso10a proteins function as sequence-specific transcription factors. Here we show that Sso10a1 and Sso10a2 exhibit different distinct DNA-binding modes. While the ability to bend DNA is shared between the two proteins, DNA bridging is observed only for Sso10a1 and only Sso10a2 exhibits filament formation along DNA. The architectural properties of Sso10a proteins suggest that these proteins fulfil generic roles in chromatin organization and compaction. As these proteins exhibit different binding behaviour depending on their DNA binding stoichiometry, altered levels of expression in the cell can be exploited to drive changes in local genome folding, which may operate to modulate transcription.

Chromatin proteins play an important role in compacting and organizing genomic DNA throughout all domains of life^1^. Besides folding the genome into a compact structure, chromatin proteins are involved in important cellular processes such as transcription, replication and repair. Although these proteins are not conserved throughout all domains of life at the level of amino acid sequence, they appear to be conserved in terms of architectural properties^1^. Chromatin proteins structure DNA by wrapping, bridging or bending the DNA helix or by forming protein-DNA filaments. Not only do the architectural properties of these proteins explain genome compaction and organization, they also provide a framework for understanding how different proteins can operate jointly to maintain a dynamic and plastic genome. The interplay of proteins with different properties is concentration dependent, which permits modulation of the degree of local and global compaction and thereby the accessibility of the genomic DNA for DNA transactions^1^,^2^.

Archaea express a diverse range of chromatin proteins that aid in organizing their genomic DNA into a compact nucleoid structure. Hence, these proteins are often referred to as nucleoid-associated proteins (NAPs). Most crenarchaea lack homologues of eukaryotic histones^3^ and synthesize other small chromatin proteins instead^4^, which is analogous to bacteria. This sets them apart from species in the euryarchaeal branch, which express true histone H3/H4 homologues that can assemble into nucleosome-like or filamentous structures^3^,^5^,^6^. Several chromatin proteins have been identified and characterized in the crenarchaeal model organism *S. solfataricus*: Cren7^7^, Sul7^8^,^9^ and Alba^10^. Cren7 and Sul7 are monomeric proteins with structural and functional homology despite a lack of sequence homology^11^,^12^. Both proteins induce rigid bends in dsDNA, resulting in compaction^13^. Alba proteins exist as dimers and display bimodal behavior, bridging DNA or forming stiff filaments along DNA, depending on protein concentration and dimer composition^14^. The balance between the two binding modes is believed to be an important mechanism in dynamic chromatin organization and gene regulation *in vivo*^14^.[Fig f1][Fig f2][Fig f3][Fig f4][Fig f5]

The Sso10a proteins of *S. acidocaldarius* are members of a family of small (10 kDa) DNA-binding protein in crenarchaea^15^. The structure of Sso10a1 reveals that it dimerises in solution via the formation of an anti-parallel coiled-coil structure^16^,^17^,^18^ (see Fig. 6). The domain at the end of the coiled-coil is believed to be involved in DNA-binding as it adopts a winged helix-turn-helix (wHTH) motif. This motif is a subfamily of the helix-turn-helix (HTH) motif that is common in DNA-binding proteins throughout all domains of life^19^,^20^. In archaea, the HTH motif is often found in transcription regulators that bind to specific sequences^21^, which might imply that Sso10a proteins have similar DNA binding properties and function. The wHTH motif generally consists of three α-helices (α1-α3) and a β-sheet wing. The α3-helix is often referred to as the recognition helix as it binds DNA by inserting in the major groove and is thought to be important for sequence specific binding. In many cases the β-sheet wing makes additional protein-DNA contacts by binding along the phosphate backbone^22^. Visualization of Sac10a-DNA complexes using electron microscopy demonstrated that Sac10a can hold two DNA duplexes together^23^, suggesting a role in chromatin organization and compaction. Little is known about the DNA-binding properties of Sso10a, besides that Sac10a binds preferentially to poly(dAdT) dsDNA (compared to poly(dA)-poly(dT) and DNA isolated from *E. coli*) with high affinity in the nanomolar range^17^. By analogy with another HTH DNA-binding protein (γδ-resolvase), it has been proposed that Sso10a binds along the DNA via the two α3-helices that are inserted in the major groove. The wing and the N-terminus could interact with minor grooves^16^. According to this model, binding of Sso10a induces bending of the DNA by ~60°. Three Sso10a homologues are encoded on the *S. solfataricus* genome: Sso10a1, Sso10a2 and Sso10a3 (29–39% sequence identity and 55–62% sequence similarity, see Fig. 1). A notable difference in the amino acid sequence of the three Sso10a proteins is that Sso10a2 contains four extra residues within the winged loop domain, not present in Sso10a1 and Sso10a3. Although nothing is known about the abundance of these proteins in the cell, the *S. solfataricus* transcriptome^24^ reveals that the transcription level of the three Sso10a proteins together is similar to that of Cren7, which constitutes ~1% of the total cellular protein^7^. This observation suggests that also Sso10a proteins are abundant within the cell. It is unclear whether Sso10a proteins primarily act as transcription regulators involved in specific gene expression or rather as chromatin proteins involved in genome compaction and organization.

For a better understanding of the architectural properties and function of Sso10a proteins, we investigated the effects of Sso10a1 and Sso10a2 proteins on DNA conformation using single-molecule micromanipulation and imaging techniques. Our data indicate that Sso10a1 and Sso10a2 exhibit different DNA binding modes, bending or bridging DNA and/or forming stiff protein-DNA filaments without apparent sequence specificity, compatible with a role as chromatin proteins. Moreover, the crystal structure of Sso10a2 as solved in this study provides a structural mechanistic basis for the differences in architectural properties of the two proteins.

## Results

### DNA compaction and decompaction by Sso10a1 and Sso10a2

Tethered particle motion (TPM)^25^, enables observation of structural changes in DNA at a single-molecule level. We used this technique to characterize the architectural properties of Sso10a1 and Sso10a2. In TPM the excursion of a bead tethered to a DNA molecule is expressed in terms of a root mean square distance (RMS). The RMS distance is affected by the conformational state of the DNA molecule. Under our experimental conditions the RMS for bare DNA is 161.8 ± 0.7 nm (see Fig. 2). In these buffer conditions, Sso10a1 and Sso10a2 bind DNA with similar affinity (K_D_ ≈ 1 nM) in bandshift assays (data not shown). Binding of either Sso10a1 or Sso10a2 reduces the RMS in a concentration dependent manner at concentrations ≤100 nM (see Fig. 2) to a minimum of 115.8 ± 0.6 nm for Sso10a1 and 120.0 ± 0.8 nm for Sso10a2 at a concentration of 50 nM. The observed reduction in RMS indicates that the DNA is compacted to a similar extent due to binding of both Sso10a1 and Sso10a2. Compaction is attributed to bending and/or bridging of the DNA.

Binding of Sso10a2 however leads to an increase in RMS for concentrations ≥1000 nM up to a maximum of 173.0 ± 3.7 nm at 3000 nM, which is higher than the RMS value found for bare DNA. The increase of RMS at high concentrations of Sso10a2 suggests that, besides bending and/or bridging, Sso10a2 has a second binding mode which leads to the formation of stiff protein-DNA filaments. DNA stiffening can occur when proteins exhibit side-by-side interactions promoting filament formation^26^,^27^,^28^. To investigate the mechanism and physico-chemical basis of the stiffening observed for Sso10a2, we investigated the sensitivity to a change in salt concentration. Reduction of the salt concentration (20 mM instead of 100 mM NaCl) shifts the concentration regime of this binding mode, while the intrinsic binding affinity is not affected; the onset of DNA compaction occurs at similar protein concentrations (see Fig. 2). At reduced salt concentration, the RMS increases beyond Sso10a2 concentrations ≥200 nM up to a maximum value of 185.8 ± 1.8 nm at 2000 nM. The effect of NaCl concentration on the stiffening mode suggests that this binding mode is determined by electrostatic interactions between neighboring proteins.

In the case of Sso10a1, concentrations >100 nM result in all DNA tethers or beads being stuck to the flow cell surface, which makes it impossible to collect data at these concentrations. It is possible that the DNA is compacted so strongly at these concentrations that bead-surface or DNA-surface interactions are promoted. Therefore it cannot be determined from these experiments whether Sso10a1 stiffens DNA at these concentrations analogous to Sso10a2.

**Figure 1 f1:**
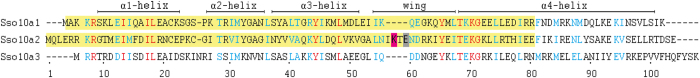
Amino acid sequence alignment of Sso10a1 (SSO10449), Sso10a2 (SSO2827) and Sso10a3 (SSO2986). Sequence alignment was performed using the Clustal Omega program^59^. Secondary structure elements are indicated based on the Sso10a1 structure. Identical residues are indicated in red and conserved residues are indicated in cyan. The DNA binding domains Sso10a1_DBD and Sso10a2_DBD are highlighted in yellow. Residues K57 and E59 of Sso10a2 are highlighted in magenta and grey respectively.

To investigate whether the observed compaction is due to the action of single DNA-binding domains or the joint action of two DNA-binding domains at the ends of the dimer, we generated truncated Sso10a proteins (see Material and Methods). As these proteins (referred to as Sso10a1_DBD and Sso10a2_DBD) only consist of the DNA-binding-domain (see Fig. 1) they are expected to be monomers in solution. Although there are some small differences in binding affinity, both Sso10a1_DBD and Sso10a2_DBD bind to DNA with a binding affinity in the same order of magnitude as that of the full-length proteins as determined by Electrophoretic Mobility Shift Assay (see SI Figure S1).

However, in TPM experiments with Sso10a1_DBD and Sso10a2_DBD no effects on the RMS are observed, not even at protein concentrations two orders of magnitude higher than those that result in maximum compaction for full-length Sso10a1 and Sso10a2 (see Fig. 3). This observation indicates that binding of the DNA-binding domains alone does not change the DNA configuration and that both DNA-binding domains of the Sso10a dimer need to interact with the DNA for DNA compaction.

### Structural characterization of protein-DNA complexes

Our TPM experiments revealed that Sso10a1 and Sso10a2 behave differently at high protein concentrations. To characterize the binding modes of Sso10a1 and Sso10a2 in this regime, we visualized protein-DNA complexes using atomic force microscopy (AFM). We used a single-nicked circular DNA substrate of 2.7 kilobasepairs (see Materials and Methods). Binding of Sso10a1 at concentrations of 0.5 μM and 1 μM (60 bp:protein and 30 bp:protein) results in compaction of the DNA molecules (see Fig. 4). Within individual Sso10a1-DNA complexes regions of DNA are aligned, which points to bridging of two DNA duplexes mediated by the protein. These Sso10a1-DNA complexes are similar to the bridged protein-DNA complexes observed for the bacterial chromatin protein H-NS^29^ and the archaeal chromatin protein Alba^14^. Bending of DNA might also contribute to the observed compaction. However, protein-induced bending cannot be quantitatively analyzed, as the small size of the proteins does not allow the identification of individual proteins on the DNA in our AFM images. Classification of the protein-DNA complexes by visual inspection revealed that the fraction of bridged molecules increases somewhat at higher Sso10a1 concentrations (from 59% at 0.5 μM to 66% at 1 μM - see Table S3 for details). At Sso10a1 concentrations above 1 μM, protein-DNA complexes could not be deposited onto the mica surface. Possibly, high Sso10a1 concentrations lead to very strong compaction, shielding the negative charge of the DNA and preventing stable interaction of the protein-DNA complexes with the mica surface. Strong compaction of DNA molecules at high Sso10a1 concentrations could also explain why protein-DNA complexes were stuck on the flow cell surface in our TPM experiments (see above).

In contrast, addition of Sso10a2 at comparable concentrations yields complexes distinctly different from those observed for Sso10a1. While addition of 0.5 μM and 1 μM Sso10a2 (60 bp:protein and 30 bp:protein) does not induce obvious changes in DNA configuration, a protein concentration of 2 μM induces a more open conformation of the plasmids, indicating stiffening of the DNA. The round and open shape of the DNA molecules is a signature of DNA stiffening as shown in our earlier studies on the HU protein^26^,^30^. These Sso10a2-DNA complexes are similar to the protein-DNA complexes observed for bacterial chromatin protein HU, which stiffens DNA at high concentrations by cooperative side-by-side binding^30^. This observation is consistent with the increased RMS values observed at high Sso10a2 protein concentration in the TPM experiments.

### Quantification of the architectural properties of Sso10a1 and Sso10a2

The observed compaction due to binding of Sso10a1 and Sso10a2 at low concentrations suggests that these proteins bend DNA. As a reduction in apparent DNA persistence length following protein binding is indicative of protein-induced DNA bending^26^,^31^, we next employed dual-trap optical tweezers to quantify the apparent persistence length upon binding of Sso10a1 and Sso10a2. Optical tweezers (OT) enable the precise mechanical interrogation of individual protein-DNA complexes^32^. We probed the mechanical response of bare DNA and protein-DNA complexes at different protein concentrations by generating force-distance (FD) curves (see SI Figs 2 and 3). We determined the DNA persistence length (L_p_) and contour length (L_c_) by extensible worm-like-chain (eWLC) model fitting of these curves. The persistence length of bare DNA is 47.2 ± 5.8 nm in good agreement with previous estimates^33^. At low protein concentrations, binding of both Sso10a1 and Sso10a2 induces softening of DNA (See Fig. 5). The apparent persistence length of Sso10a1- and Sso10a2-DNA complexes is reduced by up to 95% compared to bare DNA in the range of 20–500 nM (L_p_ is 2.5 ± 0.3 nm for Sso10a1 and L_p_ is 2.3 ± 0.5 nm for Sso10a2 at a concentration of 100 nM) (See Fig. 5). Concomitantly, the contour length of the protein-DNA complex in this regime compared to bare DNA increases by about 10% (L_c_ is 18.8 ± 0.3 μm for Sso10a1 and L_c_ is 18.4 ± 0.3 μm for Sso10a2 at 100 nM) (see Fig. 5). In the model proposed by Chen *et al*.^16^ the DNA-binding domains of the Sso10a1 dimer interact with the major groove of the DNA on a binding site of 25 base pairs, resulting in a bend of about 60°. Compared with other DNA-bending proteins (Cren7 and Sul7)^13^ resulting in similar bending, the reduction in L_p_ due to binding of Sso10a1 and Sso10a2 is remarkably high. This may be attributed to partial unwinding of the double helix, which is in agreement with the increase in contour length as well and similar to earlier observations on the TFAM protein^31^.

Beyond a concentration of 500 nM Sso10a2-DNA complexes enter a second concentration regime (exactly as observed by TPM) in which additional binding of protein returns the contour length and persistence length to bare DNA levels (L_p_ is 61.0 ± 10.5 nm and L_c_ is 16.1 ± 0.1 μm at 3000 nM). The contour length and persistence length of Sso10a1-DNA complexes remain unaltered up to the highest concentrations at which experimental data could be obtained (see Fig. 5). These results indicate that Sso10a2 stiffens DNA at high protein concentration. This is consistent with the existence of a second binding mode, in agreement with AFM and TPM experiments that were conducted at high protein concentration. Numerous other DNA-binding proteins (Alba^34^, TrmBL2^35^, H-NS^27^,^29^,^36^, HU^26^) are also known to exhibit distinct DNA binding modes in different protein concentration regimes.

In the case of Sso10a1, most all (93%) of the FD curves obtained in extension experiments exhibit irregular serrations *i.e.* a series of sudden abrupt changes in force and distance at concentrations ≥100 nM (see SI Figure 2). Beyond an extension of about 16 μm, at forces higher than 40 pN the FD curves no longer exhibit such peaks and overlap with the FD curve of bare DNA. The occurrence of peaks is an indication of the formation of DNA-loops caused by proteins that bridge remote segments of the DNA molecule^29^. Rupture of such bridges by the applied force results in an abrupt increase in contour length and reduction in force. The formation of loops is in agreement with AFM images of Sso10a1-DNA that show small patches of bridged DNA (see Fig. 4). Bridges are formed in a concentration dependent manner and are typically observed only at concentrations ≥100 nM. The observation of small loops using OT and of small bridged patches by AFM suggests that DNA bridging by Sso10a1 is not strongly cooperative. This behavior is distinctly different from the archaeal DNA-bridging protein Alba, which exhibits cooperative bridging resulting in extended bridged filaments^14^, which can withstand much higher forces (up to 400 pN) than Sso10a1-DNA complexes^14^. DNA bridging by Alba is attributed to dimer-dimer interactions^14^, based on the crystal contacts in the Alba-DNA co-crystal structure^34^. Analysis of the Sso10a1 crystal structure^16^ using the Protein Interfaces, Surfaces and Assemblies (PISA) software^37^ does not reveal crystal contacts that could mediate dimer-dimer interactions in solution and explain DNA bridging by Sso10a1. Notably, Sso10a1 increases the force at which DNA overstretches (the overstretching plateau^33^) upon protein binding (see SI Figure S2). These observations suggest that Sso10a proteins stabilize dsDNA against force-induced melting. The extension and retraction curves exhibit hysteresis and moreover do not overlap with bare DNA curves, suggesting that overstretching causes incomplete dissociation and/or that the protein is differently rebound, stabilizing the unwound DNA segments that are formed in the overstretching regime.

### Structural comparison of Sso10a1 and Sso10a2

To understand the structural mechanistic basis for the differences in architectural properties of the two proteins observed in our single-molecule experiments, we determined the crystal structure of Sso10a2 to 2 Å resolution and compared it to the structure of Sso10a1 (Fig. 6A,B) Similar to Sso10a1, the Sso10a2 monomer consists of a N-terminal winged helix domain and an extended C-terminal α-helix (α1 – β1 – α2 – α3 – β2 – β3 – α4 topology^16^). Dimerization occurs through an anti-parallel coiled-coil interaction of the α4-helix. The four extra amino acids at position 56–59 in Sso10a2 lead to an extended loop within the wing domain. Sso10a1 and Sso10a2 structures also differ in the orientation angle between the α3-helices (see Fig. 6C). As the relative position and orientation of the two α3-helices is predicted to have an important influence on the DNA-binding mode^38^, this could result in distinctly different DNA-binding properties for Sso10a1 and Sso10a2. Note that the Sso10a2 crystal structure suggests possible electrostatic interactions (K57-E59 and E59-K57) between the wings of adjacent dimers (see Fig. 7C). Such interactions could underlie the observed DNA stiffening and the salt sensitivity of this process (see Discussion & Conclusions).

## Discussion and Conclusions

In this work we have investigated the structure and architectural properties of two abundant DNA binding proteins from *Sulfolobus solfataricus*, Sso10a1 and Sso10a2. Sso10a1 and Sso10a2 exhibit similar behaviour at low protein concentrations: both proteins compact DNA. At high protein concentrations, however, the architectural properties of Sso10a1 and Sso10a2 diverge: both proteins adopt an alternative mode of binding either leading to bridging or to the formation of stiff protein-DNA filaments. Based on our observations we propose a model that incorporates the concentration dependent binding modes of Sso10a1 and Sso10a2 (see Fig. 7).

### Sso10a1 and Sso10a2 induce DNA bending

At low protein concentrations, both Sso10a1 and Sso10a2 bend DNA by binding parallel along the DNA (see Fig. 7). Bending is induced by the joint action of the DNA-binding domains, as isolated DNA-binding domains do not lead to compaction of DNA molecules. Many wHTH proteins bind DNA by insertion of the α3-helix into the major groove^39^,^40^. This binding permits additional interactions between the wing and the DNA backbone, which stabilize the protein-DNA complex. The joint action of the two DNA-binding domains can result in DNA bending; the exact conformation of the bend depends on the relative orientation of the two DNA-binding domains and the distance between them^38^. Analogous to other wHTH proteins, Sso10a is likely to bind and bend DNA by interactions between the α3-helix and the wing with the DNA. Alignment of the crystal structures of Sso10a1 and Sso10a2 suggests a difference in relative orientation of the DNA-binding domains (i.e. the α3-helix, see Fig. 1). Based on our findings we expect that the relative orientation of the α3-helix of Sso10a1 and Sso10a2 in solution is similar as both proteins bind DNA with similar affinity and bend DNA to the same extent.

### Sso10a1 bridges DNA duplexes at high protein concentrations

At high protein concentrations Sso10a1 dimers bridge DNA (see Fig. 7A). A plausible model is that bridging is achieved through dimer-dimer interactions via the coiled-coil domains. These domains might be able to form a tetrameric coiled-coil structure as found for several coiled-coil motifs^41^,^42^,^43^. Interactions between dimers that are bound along the DNA can then result in bridged DNA segments (see Fig. 7A). In solution multimeric forms of Sso10a1 have not been observed^16^, suggesting that such dimer-dimer interactions are only possible between Sso10a1 dimers that are bound to DNA. Interestingly, Sso10a2 does not exhibit DNA bridging activity at any of the measured concentrations. This could be due to differences in amino acid sequence of the α4 -helix of Sso10a1 and Sso10a2 (see Fig. 1), facilitating dimer-dimer interactions via the coiled-coil in the case of Sso10a1 but not in the case of Sso10a2.

### Sso10a2 stiffens DNA at high concentrations and zero force

**Figure 2 f2:**
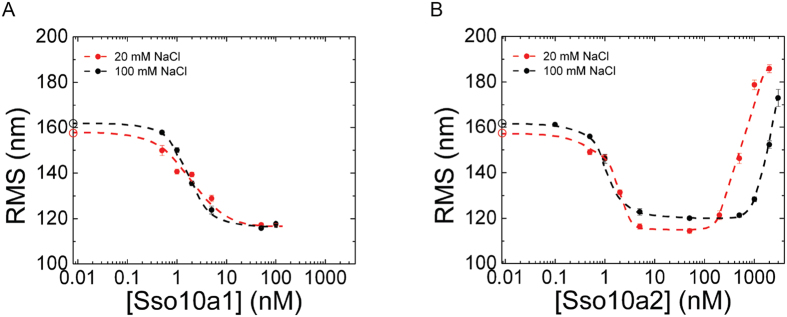
Root mean square (RMS) values as a function of protein concentration obtained from tethered particle motion experiments with (**A**). Sso10a1 and (**B**). Sso10a2 in a buffer containing 20 mM NaCl (red) or 100 mM NaCl (black). Open circles on the y-axis represent the RMS value for bare DNA (0 nM). Error bars represent the standard error of the mean. The typical RMS value of beads stuck to the flow cell surface is 45 nm. Dashed lines are a guide to the eye.

**Figure 3 f3:**
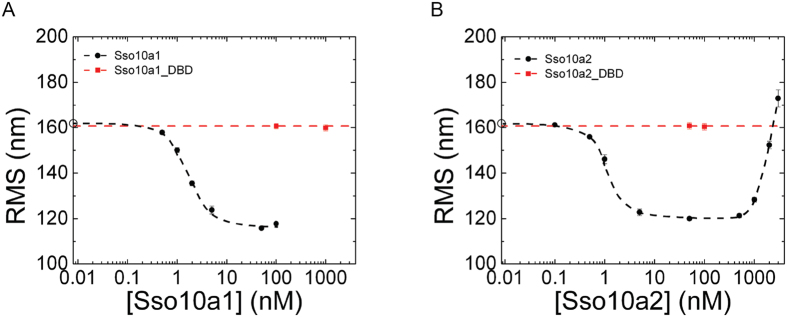
Comparison of the full-length Sso10a dimer with the isolated DNA-binding domain obtained from tethered particle motion experiments in 100 mM NaCl with (**A**). Sso10a1 (black) and Sso10a1_DBD (red) and (**B**). Sso10a2 (black) and Sso10a2_DBD (red). Open circles on the y-axis represent the RMS value for bare DNA (0 nM). Error bars represent the standard error of the mean. The typical RMS value of beads stuck to the flow cell surface is 45 nm. Dashed lines are a guide to the eye.

**Figure 4 f4:**
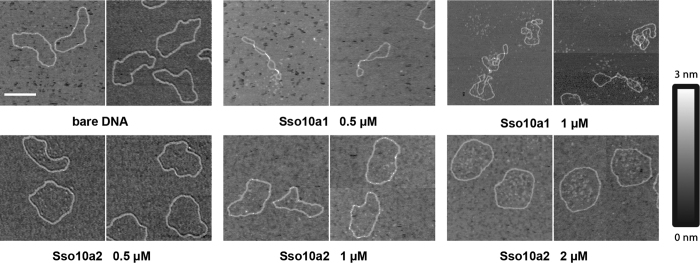
Representative images of individual Sso10a-DNA complexes, visualized by atomic force microscopy. Nicked pRD24 plasmids are incubated at different protein concentrations with either Sso10a1 or Sso10a2. Incubation with 0.5 μM and 1 μM Sso10a1 results in compacted protein-DNA complexes. Bridged patches are formed by Sso10a1. Addition of 0.5 μM and 1 μM Sso10a2 does not induce obvious changes in DNA configuration. At a concentration of 2 μM Sso10a2, the DNA plasmids obtain a more open conformation, indicating stiffening of the DNA. Scale bar represents 200 nm.

**Figure 5 f5:**
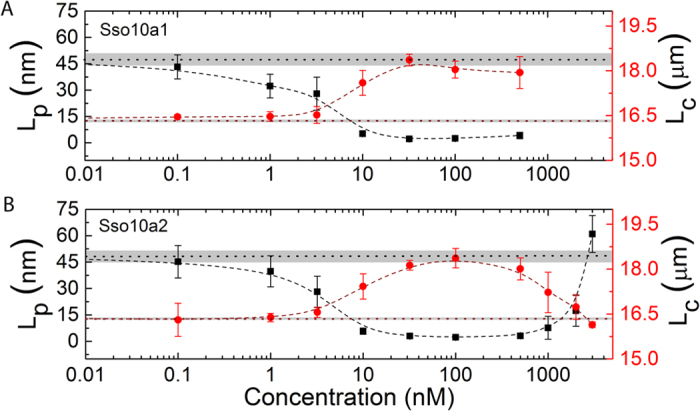
Persistence length (L_p_) (black) and contour length (L_c_) (red) values as a function of protein concentration obtained from optical tweezers experiments with (**A**). Sso10a1 and (**B**). Sso10a2 in a buffer containing 100 mM NaCl. Dashed line with grey band on the y-axis represents the value for bare DNA (0 nM) and error. Error bars represent the standard error of the mean. Thick dashed lines are a guide to the eye.

**Figure 6:**
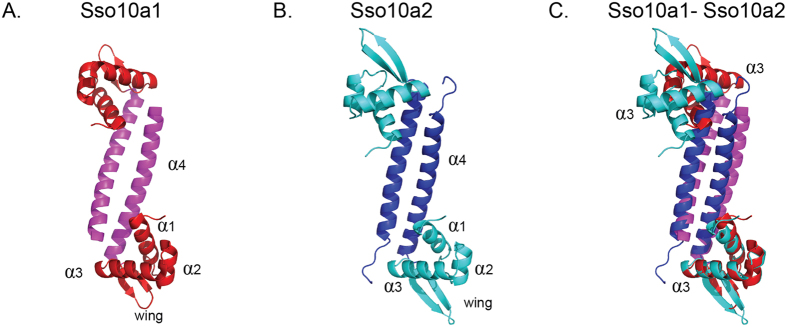
Crystal structures of (**A**). Sso10a1 (PDB code 1RJ7 (18)) and (**B**). Sso10a2 (PDB code 4WH0). The Sso10a dimer consists of an anti-parallel coiled-coil (*magenta/blue*) with two DNA-binding winged helix-turn-helix motifs (*red/cyan*) on opposite sides of the coiled-coil. The main structural differences between Sso10a1 and Sso10a2 are the length of the β-sheet wing and the relative position of the α3-helices, which are thought to be important for DNA interactions. (**C**) Sso10a1 and Sso10a2 aligned on the lower α3-helix, illustrating the difference in relative position and orientation of the two α3-helices.

**Figure 7:**
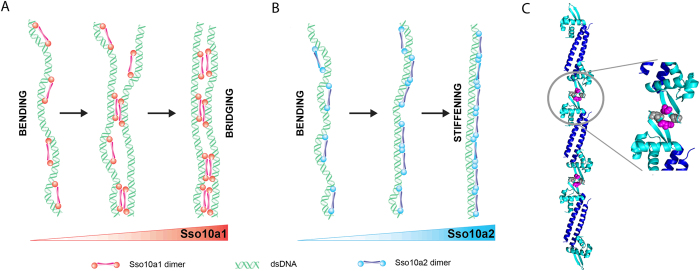
Model of the concentration dependent binding modes Sso10a. (**A**) At low concentrations, Sso10a1 bends DNA by binding in *cis*. Increased concentrations lead to dimer-dimer interactions, which bridge two DNA segments. (**B**) At low concentrations, Sso10a2 bends DNA by binding in *cis*, similar to Sso10a1. At increased protein concentrations adjacent dimers exhibit electrostatic dimer-dimer interactions via the wing, which results in protein-DNA filaments. (**C**) Sso10a2 crystal structure showing possible electrostatic interactions between adjacent dimers (PDB 4WH0). Residues K57 (magenta) and E59 (grey) within the wing domain of the dimer can form electrostatic dimer-dimer interactions when bound side-by-side along the DNA.

At high Sso10a2 concentrations, DNA is stiffened in the absence of force. Stiffening of the DNA could be due to close side-by-side binding of Sso10a2 dimers along the DNA, stabilized by dimer-dimer interactions (see Fig. 7B). As the stiffening mode is sensitive to changes in monovalent salt concentration (see Fig. 2B), it might rely on electrostatic interactions between adjacent dimers. The wing is a good candidate for interactions between Sso10a2 dimers as the wing is one of the main structural differences between Sso10a1 and Sso10a2. Moreover, the wing is positioned at both ends of the dimer, which would allow interaction between adjacent Sso10a2 dimers, assuming that the dimers bind parallel to the DNA. The Sso10a2 crystal structure indeed reveals possible electrostatic interactions (K57-E59 and E59-K57) between the wings of adjacent dimers (see Fig. 7C) that could underlie the observed salt sensitive stiffening. Interactions between the wings are not present in the crystal structure of Sso10a1 as it has a different crystal packing and has a smaller wing, lacking these residues^16^. Protein-protein interactions via the wing in a wHTH protein have previously been shown for a eukaryotic heat shock transcription factor (HSF)^44^. Unlike many other wHTH motif proteins, the wing of the DNA-binding domain of HSF is involved in protein-protein interactions rather than interacting with the DNA. Possibly, the wing of Sso10a2 is capable of mediating both types of interactions. At low Sso10a2 concentrations, the DNA occupancy is low and the wing might mostly interact with the DNA, inducing or stabilizing DNA bending. At high concentrations the wing may be involved in dimer-dimer interactions with adjacent dimers, which will favor DNA stiffening over DNA bending (see Fig. 6B).

### Multiple and different binding modes for Sso10a1 and Sso10a2

Based on our findings we conclude that the Sso10a proteins function as chromatin proteins; they bind DNA with high affinity, and display different architectural properties (DNA bending, bridging and stiffening). The relatively high protein concentrations required for our experiments do not permit detection of sequence specific binding. Determination of possible high affinity sites awaits *in vivo* genome-wide binding studies, combined with biochemical verification *in vitro* or *in vitro* optimization of DNA binding sequences using SELEX^45^. The availability of different binding modes dependent on protein concentration offers a large window in which conformational changes imposed by these proteins can be modulated. In a previous study, we proposed architectural interplay between different binding modes as a possible mechanism by which the archaeal chromatin protein Alba dynamically shapes genomic DNA^14^. Alba stiffens or bridges DNA as a function of Alba dimer composition (Alba1 homodimers or Alba1: Alba2 heterodimers) and concentration. Differential expression of Alba1 and Alba2 might thus be a mechanism to modulate global and local chromatin structure and operate to modulate gene expression *in vivo*. Other *Sulfolobus* NAPs with their own architectural modes, are expected to be able to operate synergistically. For instance, DNA bending by protein A can support the formation of small loops by a DNA bridging protein B. Such synergistic action could occur between Sso10a and other chromatin proteins such as Cren7 and Sul7 that bend DNA and/or Alba that is able to bridge DNA. Genome wide association studies on these architectural proteins – which are currently lacking – could reveal whether and how these proteins co-localize on the crenarchaeal genome and if they form cooperative multi-protein complexes as observed in bacteria.

## Materials and Methods

### Protein purification

Sso10a1 and Sso10a2 were overproduced in *E. coli* strain BL21-CodonPlus (DE3) and *E. coli* strain BL21 (DE3) respectively, containing plasmid pRD114 or pRD111 (pET11a derivatives including either the gene encoding Sso10a1(gene SSO10449) or Sso10a2(SSO2827) from *S. solfataricus*. Cells were grown in LB medium containing 30 μg/ml ampicilin at 37 °C up to OD_600_ ≈ 0.4 and expression was induced using 0.5 mM IPTG. Two hours after induction, cells were harvested by centrifugation and resuspended in 20 ml buffer A [50 mM Tris–HCl (pH 8.0), 2 mM MgCl_2_, 0.1% Triton X100, 386 μg/ml benzamidine hydrochloride and 10 mM β-mercaptoethanol]. Cells were lysed by sonication, 1000 units OmniCleave Endonuclease (Westburg) per gram cells were added and the cell lysate was incubated for 30 min at room temperature. After heating the cell lysate for 40 min at 70 °C, EDTA and NaCl were added to a final concentration of 20 mM EDTA and 100 mM NaCl. The cell lysate was centrifuged for 30 min at 37000 rpm and filtered through a 0.45 μm membrane filter (MiliPore). The supernatant was applied to a HiTrap SP column (GE Healthcare), equilibrated in buffer B [10 mM KPO_4_ (pH 7.0), 100 mM NaCl, 10% glycerol and 10 mM β-mercaptoethanol]. Protein was eluted with a linear gradient of 0.1–1 M NaCl in buffer B. After dialyzing at 4 °C against buffer B the protein was loaded onto a Heparine HP column equilibrated in the same buffer, and eluted with a linear gradient of 0.1–1 M NaCl in buffer B. Proteins were dialyzed at 4 °C against buffer B containing 200 mM NaCl, and stored at −80 °C until required.

Selenomethionine substituted Sso10a2 protein was purified as described above for the Sso10a2 protein. For overproduction, cells were grown in minimal medium containing 50 μg/ml selenomethionine. After elution from the Heparine HP column, the protein was dialyzed at 4 °C against buffer B containing 100 mM NaCl. The protein was loaded on a Resource S column (GE healthcare) and eluted with buffer B containing 1 M NaCl. Finally, the fractions containing Sso10a2 protein were loaded onto a Superdex 200 gel-filtration column (GE Healthcare) equilibrated with 20 mM NaPO4 (pH 7.3) and 150 mM NaCl. The flow rate applied to the columns was 0.8 ml/min.

The isolated His-tagged DNA-binding-domains of Sso10a1 and Sso10a2 (Sso10a1_DBD and Sso10a2_DBD) were produced by inserting the part of the gene that encodes for the amino acids M1-R73 and M1-E80 respectively with an additional His-tag at the C-terminal end into plasmid pET11a (resulting in plasmid pRD122 and pRD119, respectively). Overproduction was performed in *E. coli* strain BL21-CodonPlus (DE3) and *E. coli* strain BL21 respectively. Cells were grown in LB medium containing 30 μg/ml ampicilin at 37 °C up to OD_600_ ≈ 0.4 and expression was induced using 0.5 mM IPTG. Two hours after induction, cells were harvested by centrifugation and resuspended in 20 ml buffer C [20 mM Tris–HCl (pH 7), 500 mM KCl, 10% glycerol and 10 mM β-mercaptoethanol]. Cells were lysed by sonication, 1000 units OmniCleave Endonuclease (Westburg) per gram cells were added and the cell lysate was incubated for 30 min at room temperature. The cell lysate was centrifuged for 30 min at 37000 rpm. The supernatant was applied to a His-Trap column (GE Healthcare), equilibrated in buffer D [20 mM Tris–HCl (pH 7.0), 500 mM KCl and 10% glycerol]. Protein was eluted with a linear gradient of 0–250 mM imidazole (Sso10a1_DBD) or 0–500 mM imidazole (Sso10a2_DBD) in buffer D. Proteins were dialyzed at 4 °C against a storage buffer [20 mM Tris-HCl (pH 7.0), 10% glycerol and 10 mM β-mercaptoethanol] containing either 600 mM NaCl (Sso10a1_DBD) or 200 mM NaCl (Sso10a2_DBD), and stored at −80 °C until required. Protein concentrations were determined using a Bicinchoninic Acid (BCA) protein Assay (Thermo Scientific) and are indicated as monomer concentrations.

### Protein crystallization

Sso10a2 was concentrated to 10 mg/ml with a 3-kDa molecular weight cutoff centrifugal filter unit (Millipore). Sitting drop vapour diffusion crystallization trials were set up using the JCSG+ and PACT screens purchased from Qiagen (Hilden, Germany). The protein to reservoir solution ratio of the drops was 3:1. Crystals grown in 1 M succinic acid, 0.1 M HEPES (pH 7.0) and 0.1% w/v PEG MME 2000 were collected for X-ray diffraction analysis.

### X-ray diffraction analysis

Crystals were picked up in cryoloops and briefly soaked in a solution composed of 10% v/v glycerol in mother liquor prior to flash-cooling in liquid nitrogen. X-ray diffraction experiments were conducted on ID 14-1 at the European synchrotron radiation facility (Grenoble, France). 378 images were collected at a wavelength of 0.933 Å with an oscillation angle of 0.95° and an exposure time of 7 s per frame at 100 K. The diffraction images were processed with XDS^46^, excluding the reflections round the 2.249 Å ice-ring. Scaling and merging was done with Aimless^47^ and the Crank suite^48^ was used to obtain an initial model with the following toolchain: Afro, Crunch2^49^ and Bp3^50^ for substructure detection and refinement. Followed by Parrot^51^ for density modification and Buccaneer^52^ in combination with Refmac^53^ for model building iterated with refinement. Manual adjustments and completion of the model were performed in Coot^54^, also iterated with translation libration screw-motion (TLS) and restrained refinement in Refmac with automatically generated TLS groups and non-crystallographic symmetry (NCS) restraints. Both Parrot and all refinement cycles in Refmac were run with direct phase restraints^55^,^56^. Data reduction and refinement statistics are shown in Tables S1 and S2, respectively (see Supplementary Information). The atomic coordinates and structure factors have been deposited in the RCSB Protein Data Bank under PDB identifier 4HW0.

### DNA constructs

#### TPM and Electrophoretic Mobility Shift Assay substrate

An end-labeled DNA construct of 685-bp was obtained by PCR using biotin- and digoxygenin(DIG)-labeled primers and plasmid pRD118. pRD118 was constructed by inserting a 685-bp long fragment from the *S. solfataricus P2* genome^57^ into the NdeI and BamHI site of pET3-his^58^. The PCR product was purified using a GenElute PCR Clean-up kit (Sigma-Aldrich).

#### AFM substrate

The pRD24 plasmid (a pUC19 derivative, containing a Bpu10I recognition site inserted into the multiple cloning site) used for the AFM experiments was propagated in *E. coli* strain XL10 and purified (Qiagen plasmid midi kit) as described before^13^. pRD24 was nicked by digestion with Bpu10l (Fermentas).

#### Optical tweezers substrate

Bacteriophage λ-DNA (Roche) was biotinylated at both ends by labeling the 12-nucleotide long 5′ overhangs with dTTP, dGTP, biotin-14-dATP and biotin-14-dCTP using Klenow DNA polymerase exo^−^ (Fermentas).

### Tethered particle motion experiments

Flow cells (volume ~30 μl) were incubated with 20 μg/ml anti-DIG antibodies (Roche) and incubated for 5 min. Passivation of the surface was achieved by flushing the flow cell with 0.4% (w/v) Blotting Grade Blocker (BGB) (Bio-Rad) in buffer I [10 mM Tris (pH 7.5), 150 mM NaCl, 1 mM EDTA, 1 mM DTT, 3% glycerol and 100 μg/ml acetylated BSA (Ambion)] and incubating for 15 min at room temperature. The flow cell was flushed with buffer I, filled with 100 pM DNA solution (functionalized with biotin and DIG), incubated for 10 min and flushed again with buffer I. Streptavidin coated polystyrene beads with a diameter of 0.46 μm (1% w/v; G Kisker) were diluted 300 times in buffer I, flushed into the flow cell and incubated for 10 min to allow binding to the biotinylated-ends of the DNA. After washing the cell with buffer II [10 mM KPO_4_ (pH 6.0), 100 mM NaCl, 100 μg/ml acetylated BSA, 1 mM DTT and 0.5% (w/v) CHAPS (Sigma-Aldrich)], the desired concentration (0–2000 nM) of Sso10a1 or Sso10a2 of protein diluted in buffer II was flushed into the flow cell and incubated for 10 minutes at room temperature.

Tethered particle motion experiments were performed on an inverted Nikon microscope (Diaphot 300), using a 100x oil-immersion objective (N.A. = 1.25). Images were acquired by a CMOS camera (Thorlabs) at 25 Hz. The x- and y-coordinates of individual beads were tracked real-time by custom-developed LabView software (National Instruments) as described previously^59^. All experiments were performed at room temperature (~23 °C). Data analysis was performed as described before^13^. Each condition was measured at least twice; all experiments are independent. For each condition RMS values were obtained by fitting a single Gaussian to the histogram of the RMS values of individual tethers (N = 46–359).

### Electrophoretic Mobility Shift Assay

Electrophoretic Mobility Shift Assay analysis of protein-DNA complexes was carried out on 1% agarose gels in 0.5× TBE run at 80 V and post-stained using ethidium bromide. Sso10a-DNA complexes were formed by incubating 69 nM of the 685 bp DNA substrate (as used for TPM) with varying amounts of Sso10a proteins in TPM buffer II for 10 minutes at room temperature (~23 °C) before loading on gel.

### Atomic force microscopy experiments

Sso10a-DNA complexes were formed by incubating 100 ng of nicked pRD24 with varying amounts of Sso10a proteins in 10 μl AFM buffer [40 mM Hepes (pH 7.5) and 25 mM NaCl] for 10 min at room temperature (~23 °C). After incubation, this mixture was diluted 10-fold in 1 mM MgCl_2_ and directly deposited onto freshly cleaved mica. After 40 s, the mica disc was rinsed with HPLC water and dried with nitrogen gas. The AFM images were collected on a NanoScopeIII AFM (Digital Instruments, Santa Barbara, CA) using micro cantilevers (Olympus MCL-AC240TS-W2, resonance frequency 70 kHz, spring constant 2 N/m). Images were obtained using tapping mode in air at 2 Hz and flattened using Nanoscope software (Veeco Instruments).

### Optical tweezers experiments

DNA micromanipulation experiments were performed using dual trap optical tweezers as described. In short, two ends of DNA bind on two microspheres (1.87 μm, streptavidin-coated, Kisker) separately and the two microspheres were held in two traps, and the distance between two microspheres was determined using a custom-written LabVIEW program (National Instruments)^32^,^60^. The displacement of the trapped microsphere with respect to the trap center was detected using a position-sensitive detector. Force and distance were simultaneously recorded using a custom-written LabVIEW program at a sampling rate of 25 Hz. A multi-channel laminar flow cell was applied for capturing single DNA molecules between two microspheres in a buffer containing 10 mM Tris–HCl (pH 7.5), 200 mM NaCl, 1 mM EDTA and 1 mM DTT. Force distance (FD) curves were recorded in buffer II. DNA molecules were incubated with protein in the absence of tension (4.13 μm between both ends of the DNA molecule) for ~2 min. FD curves were generated at a pulling speed of 2 μm/s. All experiments were performed at room temperature (~23 °C). Extension FD curves were analyzed by fitting to the worm-like chain model (eWLC)^33^ for a stretching force from 0–30 pN model using custom-written Matlab data analysis software^61^. Fitting to the eWLC model yields estimates of several polymer properties: the total length (contour length L_0_) and the flexibility (persistence length, L_p_). Typical fits of the experimental data are shown in SI Figures S2 and S3. FD curves containing serrations were not fit to the eWLC model and not included in the data analysis.

## Additional Information

**How to cite this article**: Driessen, R. P. C. *et al*. Diverse architectural properties of Sso10a proteins: Evidence for a role in chromatin compaction and organization. *Sci. Rep.* **6**, 29422; doi: 10.1038/srep29422 (2016).

## Supplementary Material

Supplementary Information
