# Supplementary material for: Diverse architectural properties of Sso10a proteins: Evidence for a role in chromatin compaction and organization
Source: Sci Rep. 2016 Jul 11;6:29422. doi: 10.1038/srep29422 (PMC4941522; doi:10.1038/srep29422)
Supplement: Supplementary Information [file srep29422-s1.doc]

# SUPPLEMENTARY INFORMATION

**Diverse architectural properties of Sso10a proteins: Evidence for a role in chromatin compaction and organization**

Rosalie P.C. Driessen^1,†^, Szu-Ning Lin^1,2^, Willem-Jan Waterreus^1^, Alson L. H. van der Meulen^1^, Ramon A. van der Valk^1^, Niels Laurens^2^, Geri F. Moolenaar^1^, Navraj S. Pannu^1^, Gijs J. L. Wuite^2^, Nora Goosen^1^ & Remus T. Dame^1, *^

1. Leiden Institute of Chemistry and Cell Observatory, Leiden University, 2333 CC Leiden, The Netherlands
2. Department of Physics and Astronomy, VU University, 1081 HV Amsterdam, The Netherlands

† Current adress: LUMICKS B.V, Boelelaan 1081, 1081HV Amsterdam, The Netherlands

^*^ To whom correspondence should be addressed.

Tel: +31 715275605; Fax: +31 715274357

Email: [rtdame@chem.leidenuniv.nl](mailto:rtdame@chem.leidenuniv.nl)

**Table S1. Data collection statistics for Sso10a2 as reported by the Scala program.**

The R_merge_ factor was multiplicity weighted^1,2^.

| X-ray source | ESRF ID14-1 |
| --- | --- |
| X-ray detector | ADSC Quantum 210 |
| Wavelength (Å) | 0.9334 |
| Space group | P2_1_22 |
| Unit-cell parameters | a = 60.22, b = 69.54, c = 80.73  α = β = γ = 90.00 |
| Matthews coefficient (Å Da^-1^) | 2.31 |
| Solvent content (%) | 46.73 |
| Resolution (Å) | 40.36 – 2.00 (2.11 – 2.00) |
| Wilson plot B factor (Å^2^) | 32.24 |
| R_merge_ (%) | 3.9 (48.2) |
| R_rim_ (%) | 5.2 (54.1) |
| R_pim_ (%) | 1.4 (16.8) |
| Mean I/σ(I) | 39.6 (4.4) |
| Completeness (%) | 93.3 (95.2) |
| Multiplicity | 13.5 (9.6) |
| Anomalous completeness (%) | 92.9 (94.0) |
| Anomalous multiplicity | 7.2 (5.0) |
| Total No. of observations | 296078 |
| No. of unique reflections | 21998 |
| No. of reflections in R_free_ set | 1118 |

**Table S2. Refinement statistics for Sso10a2**

| Resolution (Å) | 40.36 – 2.00 |
| --- | --- |
| No. of reflections | 20862 |
| No. of molecules in the ASU | 3 |
| R_work_ (%) | 20.5 |
| R_free_ (%) | 24.5 |
| No. of atoms  Protein  Water | 2127  318 |
| B factors (Å^2^)  Protein  Chain A  Chain B  Chain C  Water | 45.72  37.81  45.30  54.04  43.44 |
| Root-mean-square deviations  Bond lengths (Å)  Bond angles (°)  No. of TLS bodies | 0.0167  1.7139  3 |
|  |  |
| Ramachandran favoured (%) | 98.57 |
| Ramachandran outliers (%) | 0 |
| Rotamer outliers (%) | 2.34 |

**Table S3. Qualitative classification of protein-DNA complexes visualized by AFM.** Protein-DNA complexes are classified as: ‘open’ if segments of the DNA molecule do not overlap, ‘cross-over’ if DNA segments overlap at a single crossover and as ‘bridged’ if the DNA molecule contains bridged patches of which the length is longer than a single crossover. N denotes the total number of DNA molecules counted for each condition.

| Protein | c (µM) | open | cross-over | bridged | N |
| --- | --- | --- | --- | --- | --- |
| - | 0 | 87% | 4% | 9% | 169 |
| Sso10a1 | 0.5 | 40% | 1% | 59% | 58 |
| Sso10a1 | 1 | 29% | 5% | 66% | 104 |
|  |  |  |  |  |  |
| Sso10a2 | 0.5 | 86% | 3% | 11% | 167 |
| Sso10a2 | 1 | 86% | 3% | 11% | 84 |
| Sso10a2 | 2 | 93% | 0 | 7% | 72 |

**Table S4. Physical parameters of DNA and protein-DNA complexes.** WLC fitting parameters of FD curves are shown for different protein concentrations. L_c_ represents the DNA contour length, L_p_ the persistence length. All values are the average of N molecules and the error is given as the standard deviation. Fitting parameters for Sso10a1 at 2 µM and 3 µM could not be determined (nd).

| Protein | c (nM) | Lc (µm) | Lp (nm) | N |
| --- | --- | --- | --- | --- |
| Sso10a1 | 0 | 16.4 ± 0.2 | 46.3 ± 5.8 | 105 |
|  | 0.1 | 16.5 ± 0.0 | 43.2 ± 6.9 | 10 |
|  | 1 | 16.4 ± 0.2 | 32. ± 3.0 | 16 |
|  | 3.2 | 16.5 ± 0.3 | 28.0 ± 9.4 | 18 |
|  | 10 | 17.6 ± 0.4 | 5.1 ± 1.6 | 15 |
|  | 32 | 18.4 ± 0.2 | 2.2 ± 0.3 | 7 |
|  | 100 | 18.8 ± 0.3 | 2.5 ± 0.3 | 9 |
|  | 500 | 17.9 ± 0.5 | 4.2 ± 1.9 | 14 |
|  | 1000 | 18.6 ± 0.9 | 3.5 ± 1.9 | 13 |
|  | 2000 | nd | nd | nd |
|  | 3000 | nd | nd | nd |
|  |  |  |  |  |
| Sso10a2 | 0 | 16.4 ± 0.1 | 48.1 ± 5.8 | 137 |
|  | 0.1 | 16.3 ± 0.6 | 45.2 ± 9.2 | 8 |
|  | 1 | 16.4 ± 0.1 | 39.8 ± 8.8 | 29 |
|  | 3.2 | 16.6 ± 0.2 | 28.1 ± 8.9 | 20 |
|  | 10 | 17.4 ± 0.4 | 5.8 ± 1.8 | 12 |
|  | 32 | 18.1 ± 0.2 | 3.0 ± 0.4 | 11 |
|  | 100 | 18.4 ± 0.3 | 2.3 ± 0.5 | 27 |
|  | 500 | 18.0 ± 0.4 | 3.1 ± 1.1 | 21 |
|  | 1000 | 17.2 ± 0.7 | 7.7 ± 8.8 | 19 |
|  | 2000 | 16.7 ± 0.4 | 17.3 ± 8.8 | 22 |
|  | 3000 | 16.1 ± 0.1 | 61.0 ± 10.5 | 29 |

**
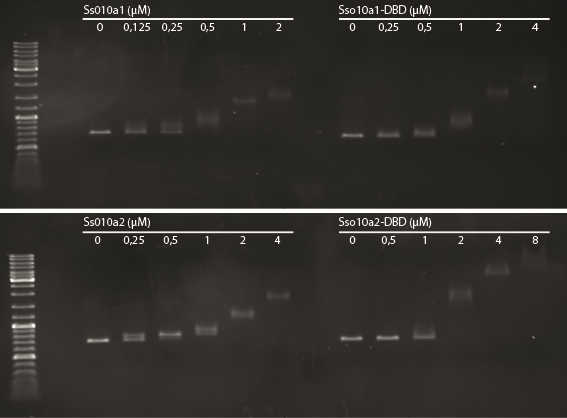
**

**Figure S1. Electrophoretic Mobility Shift Assay analysis of binding of Sso10a1, Sso10a2 and the isolated DNA binding domains Sso10a1_DBD and Sso10a2_DBD to a 685 bp dsDNA substrate**. Each series contains a titration of Sso10a1 (0, 0.125, 0.25, 0.5, 1, 2 μM), Sso10a1_DBD (0, 0.25, 0.5, 1, 2, 4 μM), Sso10a2 (0, 0.25, 0.5, 1, 2, 4 μM), or Sso10a2_DBD (0, 0.5, 1, 2, 4, 8 μM) to 69 nM DNA. The isolated DNA binding domains exhibit DNA binding with similar affinity as their full-length counterparts. GeneRuler DNA ladder was added as a reference, this contains DNA fragments of 10000, 8000, 6000, 5000, 4000, 3500, **3000**, 2500, 2000, 1500, 1200, **1000**, 900, 800, 700, 600, **500**, 400, 300, 200, and 100 bp in size.


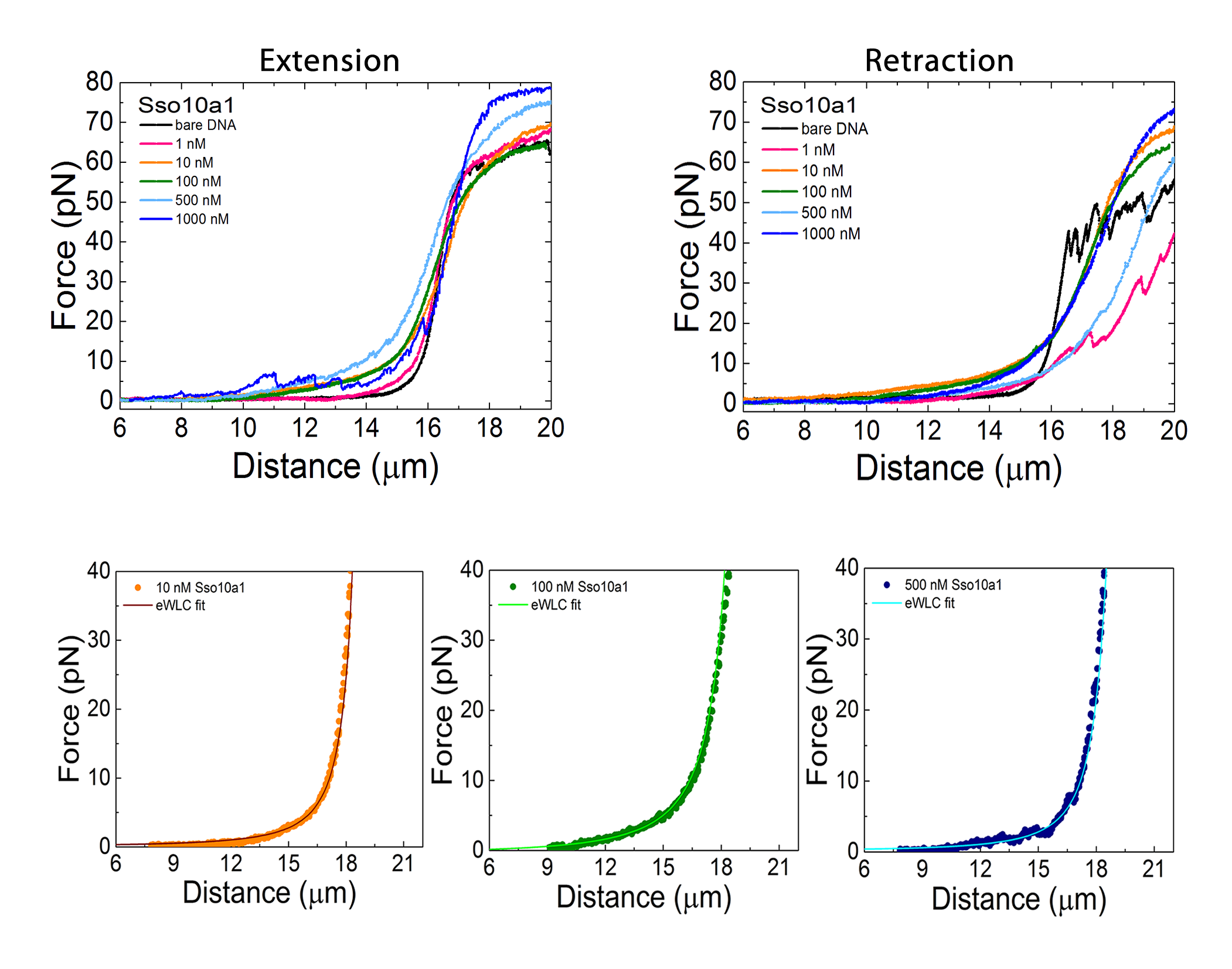
**Figure S2. Force distance curves of Sso10a1-DNA complexes at different protein concentrations (top panels) and examples of eWLC fitting (bottom panels).**


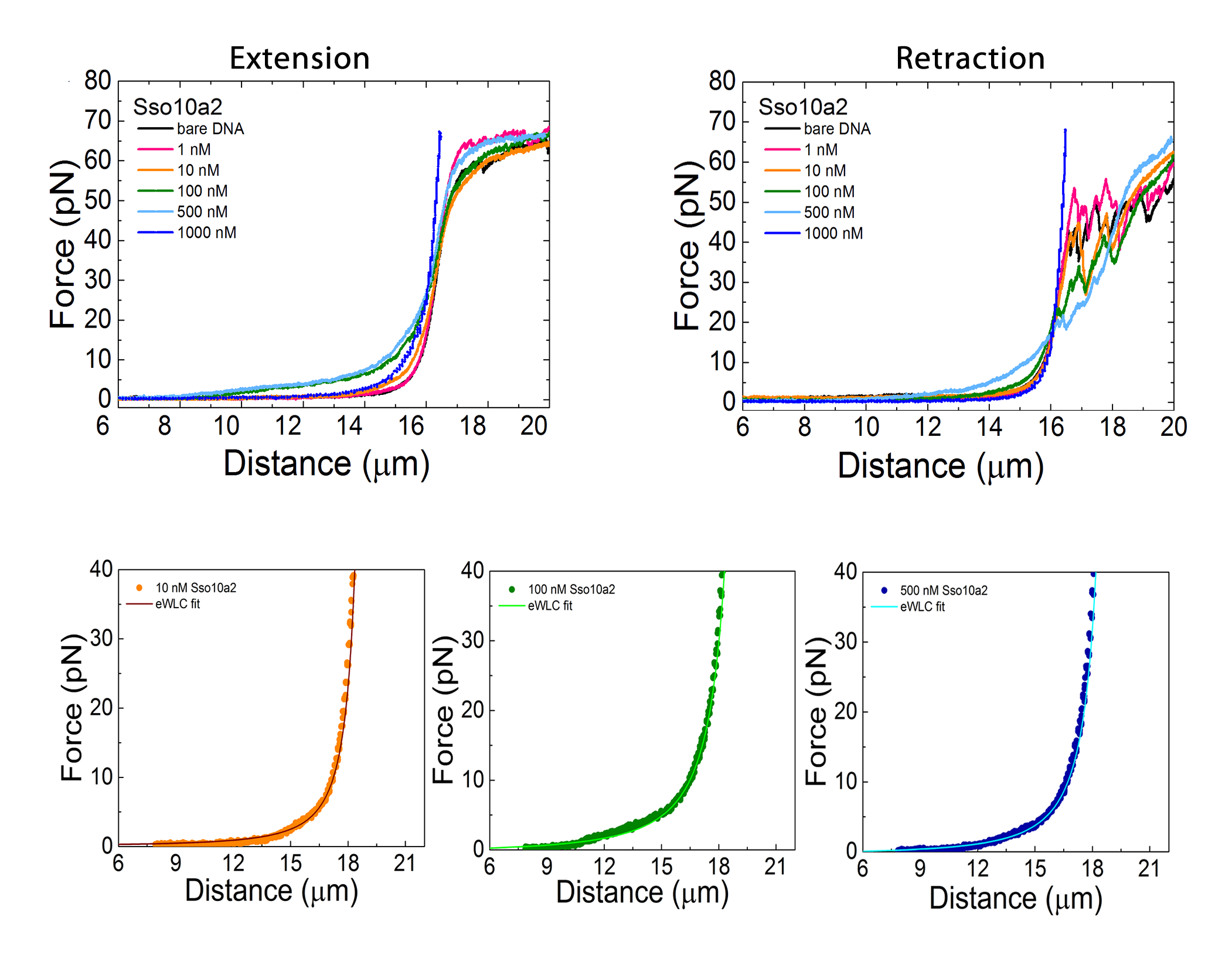
**Figure S3. Force distance curves of Sso10a2-DNA complexes at different protein concentrations (top panels) and examples of eWLC fitting (bottom panels).**

**REFERENCES**

1. Diederichs, K. & Karplus, P.A. Improved R-factors for diffraction data analysis in macromolecular crystallography. *Nat Struct Biol* **4**, 269-75 (1997).

2. Weiss, M.S. & Hilgenfeld, R. On the use of the merging *R* factor as a quality indicator for X-ray data. *Journal of Applied Crystallography* **30**, 203-205 (1997).
